# Relationship between Flooding and Out Break of Infectious Diseasesin Kenya: A Review of the Literature

**DOI:** 10.1155/2018/5452938

**Published:** 2018-10-17

**Authors:** Fredrick Okoth Okaka, Beneah D. O. Odhiambo

**Affiliations:** Department of Geography and GISc, University of Venda, Private Bag X5050 Thohoyandou 0950, South Africa

## Abstract

Flooding can potentially increase the spread of infectious diseases. To enhance good understanding of the health consequences of flooding and facilitate planning for mitigation strategies, deeper consideration of the relationship between flooding and out-break of infectious diseases is required. This paper examines the relationship between occurrence of floods in Kenya and outbreak of infectious diseases and possible interventions. This review intended to build up the quality and comprehensiveness of evidence on infectious diseases arising after flooding incidence in Kenya. An extensive literature review was conducted in 2017, and published literature from 2000 to 2017 was retrieved. This review suggests that infectious disease outbreaks such as waterborne, rodent-borne, and vector-borne diseases have been associated with flooding in Kenya. But there is need for more good quality epidemiological data to cement the evidence. Comprehensive surveillance and risk assessment, early warning systems, emergency planning, and well-coordinated collaborations are essential in reducing future vulnerability to infectious diseases following flooding.

## 1. Introduction

Flooding is the leading natural disaster in the world and one of the major environmental challenges faced by many nations in the twenty-first century [1–5]. In Kenya, like in other parts of the world, flooding has emerged as the most common and prevalent meteorological disaster [6, 7]. Some of the past significant floods in the last 20 years include the 1997/98 *El Niño* which hit many parts of Kenya causing disruption of socioeconomic activities, extensive damage to property, infrastructure, and communication facilities, and loss of life [8, 9]. In 2003, Kenya again experienced massive flooding that affected over 60,000 people; many were displaced and had to be moved into camps and provided with relief food [9, 10]. In 2006, there was another widespread flooding which also affected over 60,000 people displacing 3,500 and killing at least 7 people [10, 11]. In 2008, severe flooding affected the Rift valley region, Kitale, Transzoia, Makueni, Mwala/Kibwezi, and Budalangi areas, killing 11 people in total. Fast forward, during the first quarter of 2010, raging flash floods claimed the lives of 73 people and 1,864 livestock countrywide; over 3,375 households were displaced and 14,585 people affected in total [10]. In 2012, flash floods caused by long rains killed 84 people while displacing over 280,000 people [10, 12]. These recurring trends demonstrate the frequency and effects of floods in Kenya. Some of the worst hit areas are the low-lying regions located within the Lake Victoria basin [10]. Other areas that experience perennial floods are the coastal strip, Tana River basin, and major towns like Nairobi, Kisunu, and Mombasa City [9, 13]. Some places like Budalangi area and Kano plains found in the Lake Victoria basin experience perennial floods every year during the long and short rains [10].

Flooding in Kenya is caused by many factors and conditions. First among them are flash floods due to short intensive heavy rains, river floods as a result of rivers bursting their banks, and coastal floods mainly as a result of a combination of high tides and storms. However, increasing human interference with watersheds, drainage basins, and riparian zones has been implicated as major driving force to the recurrent flooding in many places. For example, Owuor [13] observes that floods have been known to occur in some river basins even with normal rains, which he attributes to excess surface water runoff due to poor land practices such as deforestation, land degradation upstream, and poor cultivation methods. It is apparent that both climatic and nonclimatic impacts have and are expected to influence future flooding in Kenya. On top of this, the *El Niño* phenomenon mainly driven by global warming will contribute to increased frequency of flooding in Kenya [14]. This was exemplified by the 1997/98 *El Niño* phenomenon that caused massive flooding in the country. According to Latif et al. [15], sea surface temperature anomalies in the Indian Ocean are responsible for seasonal rainfall anomalies in the East Africa region. The severity of the rainfall anomalies is stronger when the Indian Ocean Dipole coincides with *El Niño* events as happened in the 1997/98 period. The Indian Ocean sea surface temperature responds to the sea surface fluctuations in the tropical Pacific with a time lag of a few months [16]. The remote forcing from the Pacific associated with *El Niño*–Southern Oscillation (ENSO) predispose the Indian Ocean to an Indian Ocean Dipole (IOD) event via anomalous surface heat fluxes causing severe climatic conditions over the East Africa region [15, 17] leading to disastrous flooding as witnessed in 1997/98. This is set to increase in future as a result of climate change.

Flooding has a wide range of health consequences such as drowning, injury, outbreak of gastroenteritis, respiratory infections, poisoning, communicable diseases, epidemic diseases such as cholera, diarrhoea, and dengue fever, poor mental health, and disability, among others [2, 5, 18–20], but in this review, the focus is solely on infectious diseases. According to Epstein [21], three components are essential for most infectious diseases: an agent (or pathogen), a host (or vector), and transmission environment. Flooding alters the balance of the environment and often creates a conducive environment (breeding ground) for the development of pathogens and vectors. The diseases that are most likely to be affected by flooding are those that require vehicular transfer from host to host (waterborne) and or a host/vector as part of its life cycle (vector-borne) [5, 22]. In addition, flooding may hinder access and provision of urgent medical services to suppress the spread of infectious diseases leading to a wider spread. In light of the increased threat of flooding due to amplification by climate change, there is need for a better understanding of the association and underlying dynamics of outbreak of infectious diseases following flooding to inform policy [5]. This paper presents a review on the scientific evidence for the impact of flooding on infectious diseases in Kenya.

## 2. Methods

A comprehensive literature search was conducted using Google Scholar, Elsevier, and Springer Online Journals. The searched involved a combination of key words relating to flooding events and infectious diseases in Kenya. Studies were eliminated that did not focus on the impacts of flood events on the outbreak of infectious diseases in Kenya. Some articles and gray literature not meeting the specific inclusion criteria, that is, did not directly address the relationship between flooding and the outbreak of infectious diseases, were incorporated to give a better contextual line.

## 3. Results

### 3.1. Waterborne Diseases

The main cause of waterborne diseases during flooding is the contamination of drinking water supply. Floods transport bacteria, parasites, and viruses into the clean water system thus leading to the outbreak of waterborne diseases [5]. It has been observed throughout the world that waterborne epidemics have peaked during the period 1980–2006 which coincides with the increasing number of flood events [5, 23]. Many studies have revealed postflood increases in cholera, nonspecific diarrhoea, cryptosporidiosis, rotavirus, and typhoid and paratyphoid [24, 25]. In Kenya, research has shown association between cholera outbreak and flooding [26, 27]. Stoltzfus et al. [27] while looking at the interaction between climatic, environmental, and demographic factors on cholera outbreaks in Kenya found that flooding was associated with an increased risk of cholera. In an article describing the trend of cholera outbreak in Kenya, Mutonga et al. [28] report that the largest outbreak occurred during the 1997 *El Niño* rains that caused massive flooding, with 17, 200 cases (annual incidence, 60.7 cases per 100,000 population) and an estimated 555 deaths (CFR, 3.2%). This trend continued throughout 1998. In overall, during the *El Niño* period, their study indicates that 33,137 cholera cases were reported with an estimated 1,549 deaths [28]. In 2009, during the October floods, another major cholera outbreak was reported with a total of 11,769 cases and 274 deaths, which was exceptionally high compared to other outbreaks reported during nonflood periods. In a study on the dynamics of cholera outbreaks in the Great Lakes region (which includes the Lake Victoria basin) Bompangue et al. [29] using a multiscale geographic information system-based approach found that cholera greatly increased during *El Niño* events accompanied by massive flooding. Similar associations were arrived at in an earlier study by Olago et al. [26] in the Lake Victoria basin. They noted that cholera peaks coincide with high-flow peaks during *El Niño* years in the months of September, October, November, and December.

In general, diarrhoeal diseases peak during higher than average rainfall and associated flooding in Kenya. During the 1997/98 disastrous *El Niño* floods, for example, more than 15,000 cases of diarrhoea and related diseases were reported in the coastal region of the country alone with Nairobi city also recording more than 250 cases [30]. An epidemiological study by Saidi et al. [31] on infectious diarrhoeal diseases in Malindi, a coastal town of Kenya, found a clear correlation between high rainfall/flooding and the increase in the number of patients with diarrhoea (*r*=0.53, *p*=0.0074). They found that during the long rainy season from April to June 1992, for example, the number of diarrhoeal cases increased drastically.

### 3.2. Vector-Borne Diseases

Often, the reproduction, development, behaviour, and population dynamics of arthropod vectors, their pathogens, and nonhuman vertebrate reservoirs are known to be affected by changes in precipitation [22]. Incidences of mosquito-borne infections are higher with increase in the rainfall amount [5]. For example, flood provides new breeding grounds for mosquitoes, and this leads to an increase in the number of mosquito-borne diseases such as Rift Valley fever, malaria, and West Nile fever [32]. In Kenya, a number of studies have linked above the average rainfall and associated flooding to increase in malaria epidemics. For example, malaria parasite prevalence data assembled by Snow et al. [33] over 40 years between 1974 and 2014 along the Kenya coast show that malaria parasite prevalence peaks during periods of abnormally high rainfall accompanied by flooding such as in 1982, 1994, and 1997/98; during these periods, incidences of malaria disease peaks. Other studies that have linked flooding to explosive epidemic malaria outbreak are those by Maes et al. [34] and Allan et al. [35]. In their study, Allan et al. [35] noted that the unprecedented virtually uninterrupted rainfall arising from the El Niño in 1997/98 led to massive flooding in the North Eastern region of Kenya and provided ideal breeding conditions for *Anopheles* mosquitoes (malaria vectors). This led to a large increase in the vector population leading to an explosive epidemic of Malaria. In Wajir County in the region, Maes et al. [34] observe that during this period, there were a large number of admissions to Wajir Hospital due to cases of Malaria and estimate between 40 and 55 cases per 1000 population weekly malaria incidence. Brown and Murray [5] report that within a short period of 4 months, between February and May 1998, a total of 23,377 malaria cases, translating to a malaria attack rate of 39% in the population, were reported in the North Eastern region. The average crude mortality was approximately 9 per 10,000 per day, and in the under-5 population, this rose to approximately 28 per 10,000 per day [34]. In September 2006, Maes et al. [34] observe again that the flooding that affected Wajir and Garissa counties created conducive conditions for mosquito breeding leading to a large outbreak of malaria in the region.

Another mosquito-borne disease that its outbreak has closely been linked to periods of heavy rains and resultant flooding is the Rift Valley fever outbreak [36]. Woods et al. [37] explain that flooding provides a favourable environment for the hatching of the primary vector and reservoir, multiple species of mosquitoes known as floodwater *Aede,* which is the primary maintenance and source of RVF that induce the disease outbreak. The infected *Aedes* mosquito eggs from the flood water hatches in the persisting stagnant water and matures into infective adult mosquitoes [38]. Several studies and reports in Kenya have indeed linked several outbreaks of RVF to periods of abnormally high rainfall and associated flooding. Anyamba et al. [39], for example, noted that that the heavy rainfall in Kenya in late 1957 and 1982 and mid-1989 preceded Rift Valley Fever virus activity. Fast forward, CDC [40] reports that during the 1997/98 *El Niño* flood period, the largest outbreak of Rift Valley fever occurred in Kenya resulting in an estimated 89,000 infections and 478 deaths. In Garisa County, Woods et al. [37] estimated that 27,500 infections occurred during this period. In the neighbouring Wajir County, Meas et al. [34] in a paper titled “Can Timely Vector Control Interventions Triggered by Atypical Environmental Conditions Prevent Malaria Epidemics? A Case-Study from Wajir County, Kenya” report that the situation was the same with the highest outbreak ever recorded. Another major outbreak of RVF in Kenya was recorded again in 2006 after a period of 3 to 4 months of abnormally high rainfall that occasioned flooding thereby enabling vector habitats to flourish [41]. Nguku et al. [42] report that during that period, 12 cases of RVF were detected in the North Eastern region of Kenya alone, 11 succumbed to the disease. Anyamba et al. [39] observe that all known RVF outbreaks in Kenya have always followed periods of abnormally high rainfall accompanied by flooding thus implicating flooding in disease outbreak.

### 3.3. Rodent-Borne Diseases

Rodent-borne diseases are also known to increase during periods of heavy rainfall and associated flooding because of altered patterns of human-pathogen-rodent contact [43]. A study by Diaz [44] on the link between flooding and rodent-borne infectious disease outbreak indicates that heavy rainfall encourages excessive wild grass seed production that support increased outdoor rodent population; at the same time, flooding forces rodents from their burrows into built environment and closer to human population, thus increasing the risk of infectious rodent-borne disease outbreak such as leptospirosis. Leptospirosis is a systemic zoonotic disease [45]. High prevalence of the disease was observed in Garisa Kenya following the 1997/8 El Niño rains [37]. The rains increased rodent population and offered contaminated flood waters for transmission of the disease. Other studies have indicated an increase in the disease outbreak in the country and linked it to environmental drivers interplaying with changes in climate especially higher than normal rainfall/flooding. A recent study by Kimari [45] in North Eastern Kenya found a prevalence rate of *leptospirosis* bacteria among rodents to be 41.8%. In the western part of the country, Cook [46] found the prevalence rate among slaughterhouse workers to be 13.4%. While in Kibera slums in Nairobi, Halliday et al. [47] demonstrated 18.3% prevalence in a study of *Leptospira*in rodents. Among Somali pastoralists in remote arid Northeast Kenya, Ari et al. [48] reported a prevalence of 25%. However, in one study in the central region of Kenya on the interacting effects of land use and climate on rodent-borne pathogens, Young et al. [49] found that although increased rainfall had strong effects on both plant and rodent community composition and increase in total abundance, there was little direct effect on the number of infected rodents. This may imply regional variations and calls for more studies in Kenya to confirm or reject the link between flooding and risk of rodent-borne diseases.

### 3.4. Possible Health Interventions

Public health interventions are very important in reducing vulnerability to infections as a result of flooding. The interventions range from those made before, during and after flooding [5]. One of the most effective health interventions to avoid the outbreak of infectious diseases resulting from flooding is to develop Early Warning Systems (EWSs) for infectious diseases by considering flooding trends [50]. This allows those at risk to either evacuate or take precautionary measures and the public health sector to sufficiently prepare for the eventualities. An example can be drawn from Botswana where EWS efforts have been successful in reducing the outbreak of malaria during floods due to effective predictions and timely anticipation leading to implementation of timely mitigation measures [51]. However, Few et al. [19] warn that an EWS is only effective if the population take the warnings seriously and acts appropriately or takes precaution. This then requires that warnings are communicated with clarity in a simple easy-to-digest language to maximise public understanding of nature of the risk and the advised response. Abeku [52] suggests that in areas where reliable early warning systems are not in place due to technical, logistical, or other reasons, stocking contingency drugs in health centres such as antimalarial drugs across areas at risk provides an alternative approach.

Improved forecast of occurrence of rainfall anomalies over the East Africa region associated with ENSO could help in reducing the severity of outbreaks of diseases such as Rift Valley fever, cholera, and malaria as happened in the 1997/98. This could be gained from understanding of ENSO's influence on tropical Indian Ocean dynamics. It has been established that the Indian Ocean Dipole and ENSO signal have a lead time of over three months, and this timing offers an opportunity for forecasting and hence prediction of occurrence of climate anomalies over East Africa [15]. According to Linthicum et al. [53], a two- to five-month lead time would be sufficient for preventive measures such as vaccinating domestic animals and pretreating mosquito habitats with insecticides.

Emergency response planning is another health intervention. Few et al. [19] explain that this should entail well-planned emergency procedures for health systems designed and established well in advance of the flooding hazard in order to provide a basis for effective health care during and after flooding. According to Jafari et al. [54], “emergency response plans should include training in identifying and management of specific potentially threatening diseases, preparing needed equipment, supplies and materials, making local backups of supplies and tools for diagnosis and treatment, and environmental health measures for disease outbreaks” (pp. 959). A good example where this played a vital role in reducing health impact due to flooding was in Mozambique where Christie and Halon [55] report that the emergency plan put in place by the health sector for the February/March floods began in earnest in November 1999 when red flag for impending flood was raised. The preparation entailed setting plans for cholera treatment and stocking health posts with adequate drugs and providing extra stocks of malaria medicine and rehydration fluids. Hand in hand with stocking drugs and other supplies, Menne and Murray [56] point out that there is also need for emergency planning of health facilities and services to ensure that drugs and other health services can be provided from these facilities within areas affected by floods.

During and after flooding, surveillance plays an important role in early identification and consequent control of infectious disease outbreaks as well as in timely management of other health issues [57]. Menne and Murray [56] equate surveillance to the early warning system for infectious outbreaks. The surveillance should involve systematic collection and analysis of data on infectious disease [19]. Patz et al. [58] observe that disease surveillance can provide precise knowledge of incidence rates of infectious diseases arising from flooding across population and geographic region. This will help in managing and control of disease as it generates information on frequency, trend, affected population, and location. Few et al. [19] advice that, in case diseases surveillance already exists, there is need for reinforcement or enhancement to target specific disease and syndromes and also to support response actions in order to reduce disease impact and risk of transmission [5, 59].

Another intervention is the rapid disease risk assessment. It should be set up by public health responders at the onset of flooding disaster in order to take note of its impacts and health needs and risks as well as to identify appropriate interventions to put in place Kouadio et al. [60]. According to the World Health Organization [61], the risk assessment should focus on risks such as interruption and contamination of safe water, sanitation and cooking facilities, inhabitable shelter facilities and resultant population displacement with overcrowding, increased exposure to disease vector and poor access to health services. Risks assessment informs decisions to protect health and wellbeing.

One of the primary public health interventions for reducing transmission of diseases in the affected community according to World Health Organization [62] is vector control. Vector control is effective in rendering the environment unfavourable for the survival, development, and reproduction of the vector [5, 63]. In the control of mosquito, indoor residual spraying and use of insecticide-treated nets can be effective in reducing the outbreak and spread of malaria and other mosquito-borne diseases [60, 64]. In the case of rodent control, collection of refuse and appropriate disposal of waste is required to discourage rodent vector breeding. Other breeding grounds such as tall grasses can be cleared around residential areas [57].

Proper and enhanced coordination and collaboration among stake holders such as between the department of public health and livestock, emergency relief providers and public health, and researchers and local community creates a condition for better handling of outbreaks of infectious diseases that follow flooding disaster. Such coordination was found to have worked very effectively in Saudi Arabia during the huge outbreak of Rift Valley fever in 2000. According to Hassan et al. [65] and Himeidan et al. [38] Saudi Arabia effectively controlled RVF from spreading further through the “One Health” strategy that integrated among other things active surveillance surveys to detect cases of RVF among humans and animals and to locate target areas for animal vaccination. This strategy involved a collaboration between Ministries of Health, Agriculture, and Water and the Ministry of Municipalities and International Organizations such as CDC, WHO, and the National Institute of Virology [38, 66]. The World Bank [67] underscores the need for multisectoral collaboration in contributing to making public health systems more effective and resilient especially in addressing the complex nature between extreme climatic events like flooding resulting from changing climate and outbreak of infectious diseases. In multisectoral collaboration, different ministries are incorporated including health, environment, agriculture, and infrastructure. These different sectors are expected not only to develop a joint action plan but also to share information on disease outbreak in real time and coordinate their responses.

Immediate intervention that targets water, sanitation, and hygiene (WASH) is needed [68]. Access to safe clean water during and after flooding helps in minimising the health impacts of floods [56]. Adequate supply of clean water to the affected population is very important as most infectious diseases are spread through dirty water. WASH kits which include soap and water purifiers must be distributed [68]. One of the most affordable, easily available, and widely used purifier in disinfecting water is chlorine. It is known to be effective against nearly all waterborne pathogens and is handy where no alternative supply of safe clean water can be obtained [54, 60]. Temporary toilets should be provided in case the existing one was washed away or destroyed by the ragging floods.

Health awareness campaigns are required to reduce health vulnerability to floods and protect public health. The main aim of the awareness campaign is to motivate action to protect health and wellbeing of the public. Thus, this should be one of the priorities for the public health fraternity in the event of impending floods, during floods, and in flood prone areas [69]. According to Brown and Murray [5], creating individual and community awareness is crucial in reducing the risk of outbreak and spread of infectious diseases following flooding. The awareness campaign must not begin during flooding but before the floods in order to prepare communities for the anticipated negative health risks during and after flooding and what precautions can be taken to avoid outbreak and spread of infectious diseases [69].

One of the most effective primary interventions of the health risks posed by flooding lies with planned adaptation strategies. IPCC [70] recognizes that flooding and other extreme climatic events are expected to increase in frequency and intensity and as such underscores the need for the development of planned adaptation strategies to deal with these risks. Planned anticipatory adaptation as recognized in the UNFCCC (Article 3.3) is aimed at reducing vulnerability of a system by diminishing the risk [70]. These include the construction of strong dykes and sea walls and fortifying sanitation systems and other infrastructures in order to prevent water from bursting the river banks and shore lines and destroying sanitation facilities thus reducing the risk of the outbreak of infectious diseases.

## 4. Discussion

The primary objective of this review was to examine the relationship between flooding and outbreak of infectious diseases in Kenya. The review presented findings from studies from 2000 to 2017. The effects of flooding on outbreak and spread of infectious diseases are imposed through impacts on pathogens, vectors/hosts, and disease transmission [71]. Flooding can cause interruption of clean water supply and sanitation, population displacement and resultant overcrowding, and increase in exposure to disease vector, all of which are a recipe to outbreak of infectious disease [5, 69]. In Kenya, flooding is a significant factor in the outbreak and faster spread of infectious diseases. Spikes in the outbreaks of Rift Valley fever, malaria, diarrheal diseases, and even leptospirosis have been documented in Kenya [26–28, 31, 33, 34, 37, 41]. But there have also been conflicting findings on the effect of above normal rainfall/flooding on rodent-borne pathogens with one showing that above normal rainfall had little direct effect on the number of infected rodents despite an increase in abundance of rodents [49].

Brown and Murray [5] underscore the importance of surveillance in order to appropriately understand the effect of flooding on infectious disease incident. Surveillance together with effective early warning systems and accurate predictions of pending flood disaster are important tools in reducing vulnerability to infectious disease. A comprehensive risk assessment could be incorporated into the surveillance to help determine priority diseases to prioritize in prevention and control [5]. In addition, well-planned emergency procedures are crucial in managing infectious diseases. Furthermore, well-coordinated collaborations are essential in reducing spread of the infectious diseases particularly where humans and livestock pathways are at play. Himeidan et al. [38] recommend that a country like Kenya that has repeatedly experienced the outbreaks of RFV should adopt the Saudi Arabian approach in the future.

From the literature review, it is noted that most of the studies that explore the relationship between flooding and the spread of infectious disease established empirical association but not a causal relationship. To obtain direct cause effect relationship, information should be obtained on health before, during, and after floods [56]. However, in the reviewed literature, data were mainly collected retrospectively and not longitudinal as to reveal clear cause-effect relationships. The cause-effect/link relationship can also be more precisely determined through randomized control trials (RCT); in fact, there is a growing belief that it is the only one that can provide perfect evidence that clearly link outbreak of infectious disease and flooding phenomenon; however, it is likely to be too expensive, requires a lot of time and man power, and could raise a lot of ethical concerns [43, 58, 72, 73].

## 5. Conclusion

Flooding plays an important role in the outbreak and the spread of infectious diseases as it creates conditions for the multiplication of pathogens and vectors. In this review, we have drawn attention to the association between infectious diseases and flooding in Kenya. The increasing frequency of flooding in Kenya therefore means that the country will experience more heightened cases of infectious diseases resulting from flooding. To mitigate infectious disease risk in Kenya, this review is an important read for those involved in planning response and recovery. However, there are still clear research needs to improve the understanding of the association between flooding and outbreak of infectious diseases in Kenya. More thorough epidemiological studies on cause-effect relationship between infectious diseases and flooding particularly using randomised control trails are urgently needed. Studies should also focus on assessing effectiveness of public health interventions that have been utilized in Kenya in minimising risk from infectious disease following flooding. There is also need to clearly document evidence on the quantification of the risk of infectious disease following flooding.
